# How to Codeliver Translational Health Service Research Education With Clinicians Through Effective Governance

**DOI:** 10.1111/tct.70104

**Published:** 2025-05-13

**Authors:** Robyn Taylor, Nazlee Siddiqui, Bridget Farrell, Sarah Ng

**Affiliations:** ^1^ School of Population Health, Faculty of Medicine and Health University of New South Wales Kensington Australia; ^2^ Australian Institute of Health Service Management, College of Business and Economics University of Tasmania Hobart Australia; ^3^ South Western Sydney Local Health District Sydney Australia; ^4^ Sydney Local Health District Sydney Australia

**Keywords:** education, governance, health service improvement, health service research, knowledge translation

## Abstract

Healthcare organisations and academic institutions are realising the value of codelivering health service translational research education serving dual purposes of (1) student research skills development and (2) health service improvements. Those initiatives are often coordinated through collaborative partnerships between healthcare organisations and universities. However, there is limited guidance for institutions about how to set up effective governance for the management of applied research education. In this paper, we draw from our collective experience of university educators and health professionals, delivering translational research education between two universities and four state‐level public health organisations or local health districts. Specifically, we present practical insights of four critical governance elements: academic and industry project management, collaborative supervision, team teaching approach and an interorganisational governance structure. These insights contribute learning on how to set up the required governance to effectively operationalise and codeliver translational research education in clinical health settings. This can assist universities and healthcare organisations to develop similar programs for quality education and impact, health workforce development, and service improvement.

## 1 Introduction

Knowledge translation and delivering applied research education remain a difficult agenda in closing knowledge–practise gaps in health [1]. Arriving at pragmatic educational approaches which serve the dual purpose of (1) research skills development and (2) health service improvements in clinical environments can be complex and challenging. At the university end, it can become resource intensive to deliver a practice‐oriented research education program with appropriate course level assessments and teaching contact hours demanded by rigorous knowledge translation for clinical and health service settings [2]. At the health organisation end, embedding and rewarding collaborative translational health service research between clinicians, health managers, health service researchers and university managers is yet not common [3]. While motivation of individual learners is necessary for translational research education (TRE), learners are often time‐poor [4] and/or lacking necessary contextual knowledge and research skills to engage in translational studies [5].

Effective governance and coordination between health organisations and academic institutions can help to manage these challenges [5]. However, there remains little guidance about how to set up these collaborative arrangements. While general frameworks exist about university–industry collaboration [6], they are often lacking in specific details about the required learning and teaching review processes followed in universities. Moreover, the general frameworks do not reflect on ways to negotiate challenges with decision‐making structures adopted for research projects in health organisations. This paper addresses that gap and offers practical guidance demonstrating how the two organisational governance processes and structures should connect for health services research education codelivery.

## 2 Health Service TRE Programs

The guidance provided in this paper came from our collective experience of university educators and health professional roles, delivering TRE between two Australian universities and four local health districts (LHDs) in New South Wales. Critical governance elements of the education programs include (1) academic and industry project management, (2) collaborative supervision, (3) team teaching approach and an (4) interorganisational governance structure. LHD staff enrol in the programs as postgraduate students and complete a translational health services research project in their second study year. The critical governance elements of the program are outlined below.

## 3 Academic and Industry Project Management

Industry research project management is facilitated through several divisions in the healthcare organisation including service improvement and research committees, senior organisational executive, research ethics committees and education and workforce development units. Similarly, a working group of academic mentors, course convenors, the discipline program lead and university learning and teaching committees provide academic management of the project. Together, collaboration across academic and industry teams facilitates industry aligned, ethical and systematic research designs for service improvement in clinical settings. That collaboration also enables students to translate their study outcomes into health service improvements and disseminate their findings for academic and industry audiences at state, national and international levels [7].

*Collaboration across academic and industry teams facilitates industry aligned, ethical and systematic research designs for service improvement.*

## 4 Collaborative Supervision

Students are assigned an academic and a health industry mentor to supervise the research project. Academic mentors supervise the research component of the projects. They provide guidance about the study design, literature review, data collection, analysis and dissemination processes. Industry mentors ground the projects to the clinical health organisational context. They provide contextual guidance and advice about the organisational need, subject topic area and participant recruitment strategies. Additionally, they test the suitability of proposed data collection methods at the organisational site and verify student's evidence informed project recommendations for clinical service improvement at the policy and practice levels [5]. Students lead their projects, coordinating meetings, resources and communication between the academic and industry mentors.

## 5 Team Teaching Approach

The academic mentors, course convenors and discipline program lead at the university collaborate as a team to develop research workshops and monitor student progress and skills development. This collaboration also includes problem‐solving any issues which may unexpectantly surface for individual students. The approach enables academics to draw on the research and disciplinary expertise of each other and foster continuous learning and innovation for research teaching practices [8]. Mentors and course convenors attend and contribute to the research workshops. They attend team meetings and communicate via email about teaching items. The team teaching approach makes it possible to provide the teaching contact hours, including assistance for the assessments, as required for a rigorous knowledge education program.

## 6 Governance Structures for Translational Health Research Education

**FIGURE 1:**
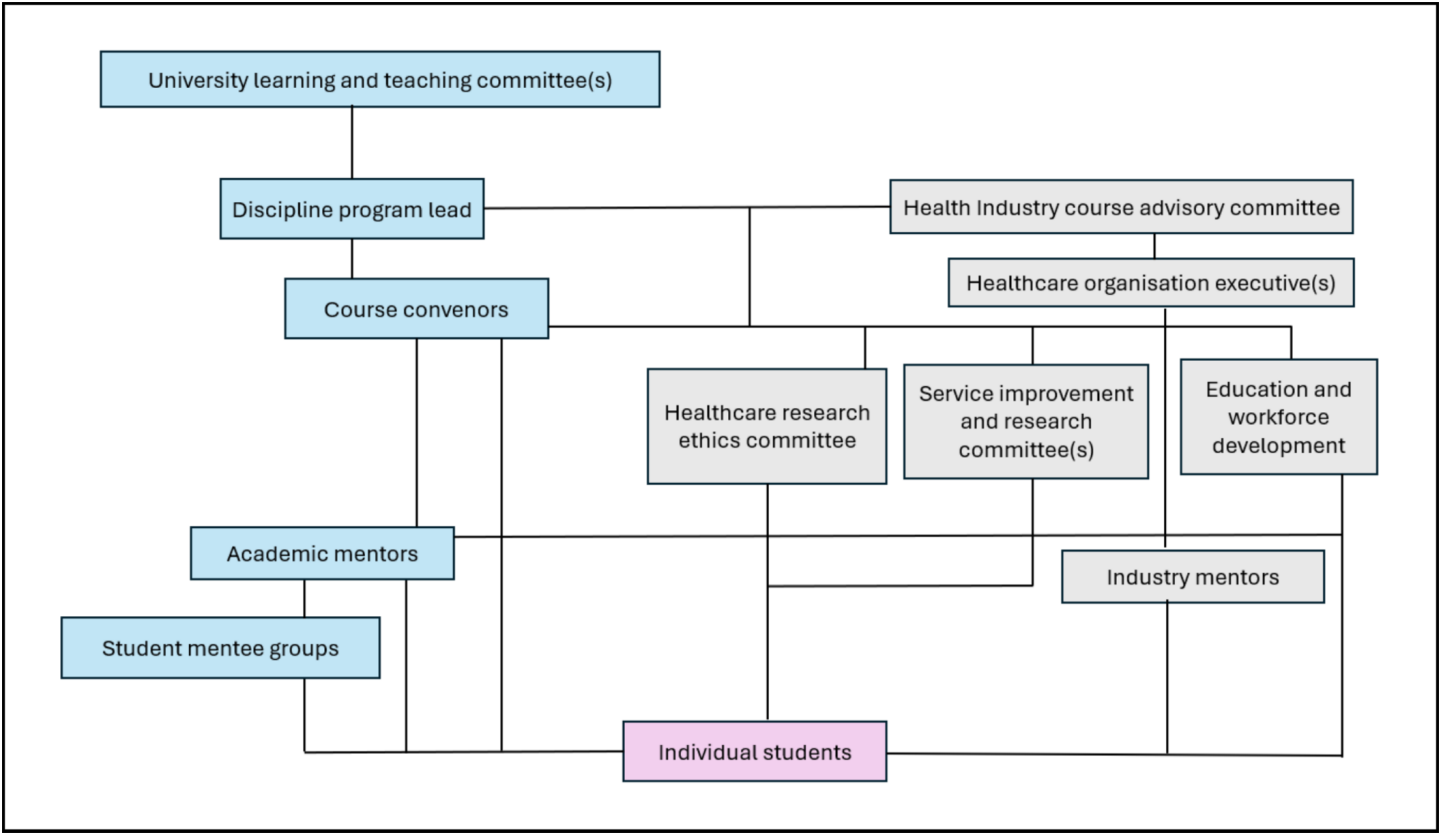
TRE program interorganisational governance structure.

While the previous three elements outlined the governance processes, this section presents the governance structures for the health service TRE program's codelivery. A program reporting and management structure is presented (Figure 1). While reporting lines have been positioned hierarchically for the university and the health organisation regarding the education program, the connections show the communication channels between stakeholders. Table 1 clarifies the stakeholder's roles and responsibilities. These items provide useful guidance tools on how to set up structural arrangements to codeliver a TRE program according to the usual decision‐making and teaching and learning systems at university and health organisations.

**TABLE 1:** Roles and responsibilities in the health service TRE program.

Role	Responsibilities
Individual students	Lead the translational research project and coordinate support from a broader team including course convenors and all other parallel and reporting stakeholders as shown in Figure 1
Student mentee groups	Facilitate research skill development and peer support through group meetings with an academic mentor Group sizes vary between 5 and 8 students each
Academic mentors	Support the research skill development of individuals in the student mentee group Maintain rapport with course convenors and stakeholders in the health organisation (as shown in Figure 1) to help students solve challenges in the research journey
Course convenors	Coordinate all TRE units and ensure their alignment to university learning and teaching policies, independent program accreditation requirements and healthcare organisation needs Foster collaboration between academic mentors to problem‐solve any individual research issue
Discipline program lead	Ensures university strategic vision is operationalised in alignment to the objectives of the partnership agreement with healthcare organisations Manage the strategic relationship with a health industry course advisory committee that represents multiple health organisations Provide overall university program oversight at a discipline level Line management to course convenors
University learning and teaching committee	Assures alignment of the TRE units with university learning and teaching strategies, policies and best practices Reviews quality and impact of TRE units and assures the education units comply with independent program accreditation requirements
Industry mentors	Support an individual student obtain a contextual understanding of their research topic in their organisation Feasibility test data collection plans against organisational resourcing Support an individual student to develop realistic, achievable, feasible recommendations from their research
Healthcare research ethics committee	Reviews student research ethics applications to ensure they comply with national ethics guidelines Manage site governance approvals for research implementation at the health organisation's location
Service improvement and research committees	Provides input to shape individual student research questions in alignment to the health organisation strategic and research goals Provides support and oversight to implement students' research in clinical service areas
Education and workforce development	Coordinates the promotion of the university course in the health organisation Ensures alignment between the university course and the education and training needs of the health organisation's workforce
Health service organisation executive(s)	Ensures that the health organisation's strategic vision is operationalised through the partnership with the universities Partners with the college/school discipline program lead regarding the strategic vision of the translational program Ensures top management support to implement students' research in clinical service areas
Health industry course advisory committee	Representative of multiple health organisations highlighting the need for translational research skill attainment in the industry Reviews TRE programs against local, state and national strategic health needs for now and the future

Meetings, communication and collaboration occur as required to manage the program within the academic teaching calendar. While Table 1 shows each stakeholder's usual responsibilities in the program's codelivery, these responsibilities are open to continuous refinement as the case is with any knowledge translation initiative.

Box 1 describes how the above explained critical governance elements are put in practice taking a practical research scenario.

Box 1 **Application of critical governance elements in a practical scenario.**

***The scenario:***

An LHD is investigating how to enhance quality of virtual care delivery for allied health services through workforce capability development. This initiative is aligned with the LHD's strategic direction about better access to care, providing digitally enabled services, greater staff engagement and safe care across all setting. A student in the health service TRE program who is also a staff member in the LHD will drive this investigation.

- ***Academic and industry project management:***

The project management of this investigation is guided by the TRE program interorganisational governance structure (Figure 1). The LHD health service organisational executives in collaboration with stakeholders identified in Figure 1 decided on a mixed methods design (focus group discussions and secondary quantitative organisational data) and specific research question, that is, what are essential workforce capability areas to enhance quality of virtual care delivery.

- ***Collaborative supervision:***

The student progresses their research project, under supervision of the academic and industry mentors, within the context of a broader organisational (e.g., health research ethics committee) and student mentee group. Examples of specific issues resolved through this supervision are customising the ethics application to fit low and negligible risk level, the academic mentor facilitating the focus group discussions to manage organisational bias and the industry advisor identifying therapists from physio, occupational and speech as suitable research participants.

- ***Team teaching approach:***

Through the team approach between academic mentors, course convenors and the discipline program lead, six workshops were delivered in a year. These workshops covered topics common to all students, for example, aligning a research question to methods, developing an ethics and site‐specific application, research rigour, data collection, data analysis and research dissemination. Each student, including the student driving this investigation, also received individualised consultation from the academic and industry mentors about developing and presenting pragmatic research recommendations.

## 7 Conclusion

This paper provides practical guidance on how to codeliver a health service TRE program according to both university and health organisational governance processes. It is important that individuals and units in both organisations become boundary spanners and function as a multidisciplinary team within interorganisational governance elements, achieving dual purposes of staff research skill development and health service improvements. Stakeholders in the health service TRE program must also make a commitment to continuous improvement in the program codelivery. This can ensure the programs value continuity in achievement of the dual purposes against any policy and practice changes in academic, health or clinical contexts.

## Author Contributions

**Robyn Taylor:** conceptualization, writing – original draft, writing – review and editing, methodology, formal analysis. **Nazlee Siddiqui:** conceptualization, writing – original draft, writing – review and editing, methodology, formal analysis. **Bridget Farrell:** conceptualization, writing – original draft, writing – review and editing, methodology, formal analysis. **Sarah Ng:** conceptualization, writing – original draft, writing – review and editing, methodology, formal analysis.

## Data Availability

Data sharing not applicable to this article as no datasets were generated or analysed during the current study.
